# Lessons from the COVID-19 Pandemic: Promoting Vaccination and Public Health Resilience, a Narrative Review

**DOI:** 10.3390/vaccines12080891

**Published:** 2024-08-07

**Authors:** Flavia Pennisi, Cristina Genovese, Vincenza Gianfredi

**Affiliations:** 1 Faculty of Medicine, University Vita-Salute San Raffaele, 20132 Milan, Italy; 2 Department of Biomedical and Dental Sciences and Morphofunctional Imaging, University of Messina, 98125 Messina, Italy; 3 Department of Biomedical Sciences for Health, University of Milan, Via Pascal 36, 20133 Milan, Italy

**Keywords:** vaccine hesitancy, public health strategy, COVID-19 pandemic

## Abstract

The COVID-19 pandemic has underscored the critical importance of adaptable and resilient public health systems capable of rapid response to emerging health crises. This paper synthesizes the lessons learned from the COVID-19 vaccination campaign and explores strategies to enhance vaccine uptake in the post-pandemic era. Key challenges identified include logistical, economic, sociocultural, and policy dimensions that impact vaccination efforts, particularly in low-resource settings. The analysis highlights the need for resilient supply chains, effective communication, community engagement, and equitable access to healthcare resources. The rapid development and deployment of mRNA vaccines exemplify the potential of innovative vaccine technologies, though public trust and acceptance remain crucial. Strategies such as partnerships with local leaders, tailored messaging, and integration of digital tools are essential for combating vaccine hesitancy. By applying these insights, future vaccination campaigns can be more efficient, equitable, and resilient, ultimately improving public health outcomes globally. This paper aims to inform policy and practice, ensuring that public health strategies are evidence based and context specific, thus better preparing for future health challenges.

## 1. Introduction

Vaccination is a cornerstone of modern public health strategies, effectively controlling and, in some cases, eradicating, infectious diseases that once posed severe threats to global health [1]. The introduction of vaccines has historically led to dramatic reductions in morbidity and mortality associated with infectious diseases such as smallpox, polio, and measles [1]. However, the emergence of the COVID-19 pandemic has presented unprecedented challenges and complexities to global health systems, vaccination campaigns, and public perception of vaccines.

The global spread of COVID-19 highlighted critical vulnerabilities in public health infrastructures and exposed significant disparities in health resource distribution and accessibility [2]. Countries with limited healthcare resources faced overwhelming challenges, as the pandemic strained systems already burdened by underfunding and under-staffing [3]. The pandemic response necessitated rapid development, approval, and deployment of vaccines at a scale and speed previously unencountered [4]. Within a year of the virus’s identification, several vaccines were authorized for emergency use, a process that typically takes a decade or more [5]. This rapid advancement, while showcasing scientific achievement, also engendered various degrees of vaccine hesitancy and skepticism fueled by misinformation, rapid changes in health guidelines, and political polarization [6]. Furthermore, the post-COVID-19 era is characterized by an evolving landscape of vaccine technology and delivery, where mRNA vaccines have come to the forefront, offering new modalities for rapid vaccine development [7]. This technological leap, while promising, requires public trust and acceptance to be effective in managing public health crises.

The ongoing necessity to promote vaccination uptake remains critical, not only for COVID-19 but also for other preventable diseases. Public health efforts must now navigate the compounded challenges of restoring public confidence, combating misinformation, and adapting strategies to diverse and changing social contexts [8]. The post-COVID-19 era presents an opportunity to build on the lessons learned during the pandemic to strengthen public health systems, enhance global cooperation in health emergencies, and develop more resilient strategies for future challenges [9]. The advancements in vaccine technology introduced during the pandemic have set a new standard for rapid and adaptable disease response, potentially transforming global health security for the better.

This paper aims to synthesize the lessons learned during the COVID-19 pandemic regarding vaccination promotion and to explore effective strategies for increasing vaccine uptake in this new era. By doing so, it seeks to provide actionable insights that can inform policy and practice, ultimately improving public health outcomes globally.

## 2. Methods

A comprehensive literature search was conducted to identify relevant studies on vaccination and public health resilience in the context of the COVID-19 pandemic. The following electronic databases were searched: PubMed, Scopus, Web of Science, and Google Scholar. The search included articles published from January 2020 to February 2024 in English. Both peer-reviewed articles and preprints were considered to ensure the inclusion of the latest research findings. The search terms used included combinations of keywords and phrases such as “COVID-19 vaccination”, “vaccine uptake”, “vaccine hesitancy”, “public health strategies”, and “mRNA vaccines”. Boolean operators (AND; OR) were used to combine terms and refine the search results. Studies were included if they met the following criteria: (i) focused on COVID-19 vaccination strategies, challenges, and outcomes; (ii) discussed public health strategies related to vaccination and public health resilience; (iii) included data on measures to combat vaccine hesitancy; and lastly, (iv) articles published in English between January 2020 and February 2024.

## 3. COVID-19 Vaccine Hesitancy: Addressing Challenges beyond the Pandemic

Vaccine hesitancy was a pivotal challenge in the public health response to the COVID-19 pandemic, undermining efforts to achieve herd immunity and control the spread of the virus [8]. This reluctance or outright refusal to receive vaccination, despite the availability of efficacious vaccines, is rooted in a complex amalgamation of factors. These include widespread misinformation, deep-seated distrust towards pharmaceutical entities, government agencies, and the healthcare infrastructure, as well as varying philosophical, religious, and socio-cultural objections [10,11]. The rapid development and approval of COVID-19 vaccines, though a remarkable achievement in medical science, have paradoxically fueled hesitancy among certain segments of the population due to concerns over perceived rushed testing and potential adverse effects [12].

For instance, pregnant and breastfeeding women have shown unique challenges in vaccine acceptance due to fears of teratogenic effects or other adverse outcomes that might affect their offspring [13]. Such concerns are magnified by the novel mRNA technology used in some COVID-19 vaccines and the accelerated pace of vaccine development, which may be perceived as insufficient to ensure safety and efficacy [14]. 

Public trust was tested by the speed of vaccine rollout and the evolving nature of scientific guidance, which often led to confusion and misinformation [15]. The implementation of mRNA vaccine technology, notable for its rapid adaptability and development speed, further exemplifies this challenge as its acceptance is critically dependent on public trust [7]. Addressing these issues comprehensively requires a multifaceted approach: this should encompass the deployment of targeted communication strategies, the enhancement of transparency in vaccine development and safety protocols, and the bolstering of health literacy through community-based educational initiatives [16,17]. In this perspective, frontline health workers (FHWs) play a pivotal role in combating vaccine hesitancy through direct patient engagement and are crucial in the dissemination of clear, evidence-based information about the benefits and risks associated with vaccines [18]. Their interaction with patients—providing reassurance, clarifying misconceptions, and explaining the scientific underpinnings of vaccine recommendations—is essential for enhancing vaccine literacy and acceptance [19]. Moreover, the integration of digital tools is fundamental in supporting vaccination programs and plausible reducing vaccine hesitancy [20]. An example could be the integration of Immunization Information Systems (IIS) into vaccination programs that significantly aid these efforts by maintaining up-to-date immunization records, facilitating timely and personalized outreach, and monitoring vaccine efficacy and safety [21,22]. The integration of comprehensive IIS is essential in the strategic management of vaccine hesitancy [23]. By improving the accuracy and availability of vaccination data, IIS enhance the efficacy of public health interventions aimed at increasing vaccine uptake [24]. Addressing the roots of vaccine hesitancy through a combination of confidence-building, convenience enhancement, and targeted communication strategies can significantly reduce barriers to vaccination and lead to higher immunization rates.

Only through such sustained and inclusive efforts can the public’s confidence be restored and the full potential of vaccination campaigns realized in managing public health crises.

## 4. Enhancing Vaccine Acceptance: Effective Strategies for Public Health 

The necessity of designing effective interventions for a broad range of target audiences has become even more pressing during the COVID-19 pandemic due to the accelerated dissemination of misinformation. The global pandemic has facilitated the advancement and realization of novel strategies to encourage vaccine uptake, offering valuable lessons applicable to other vaccination programs. The strategies that have proven effective can be grouped into six broad categories: collaboration and trust, communication and information, countering misinformation, supporting individuals transitioning from uncertainty to vaccination, improving access, and mandates and incentives [25]. 

The establishment of collaborative relationships with local community leaders has proven to be an effective strategy for enhancing access to and uptake of the COVID-19 vaccine [26]. The relationships between primary care providers, other trusted healthcare providers, and community leaders have facilitated the implementation of creative solutions that align with local preferences and needs [27]. Collaboration with community organizations, faith leaders, and local influencers has been instrumental in building trust and promoting acceptance in underserved communities. The empowerment of community members as advocates and educators has facilitated the engagement of individuals who are hesitant about vaccination and addressing misinformation. For example, in Indonesia, this community-driven strategy has significantly contributed to increasing childhood immunization coverage from 80% to 94.6%, exceeding the national target of 94.1% [28]. 

Another strategy that has proven effective is to focus on the dissemination of information about the importance and advantages of vaccination. Emphasizing the social and ethical responsibility of vaccination to protect oneself, loved ones, and the broader community has fostered pro-vaccination attitudes. Information regarding the efficacy of vaccination programs and the tangible benefits of vaccines in preventing disease and saving lives has reinforced the positive aspects of vaccination. In Indonesia, targeted public health campaigns resulted in an increase in vaccine uptake by 10% within six months. Additionally, surveys showed a 15% reduction in the number of people who believed in common vaccine myths after these campaigns were launched [29,30]. Similarly, the inclusion of a diverse range of voices, including those from the community, clinicians, scientists, and government agencies, has enabled individuals to see themselves represented in vaccination campaigns [31]. Information about vaccination sites has been disseminated through a variety of channels, including television, radio, and social media, as well as through local community networks. Personalized text interventions and appointment reminders, such as notifications about vaccine availability, have been shown to increase vaccine uptake by 6% among American adults [32].

The advent of social media has provided a valuable avenue for disseminating information to specific target audiences. However, it has also facilitated the proliferation of highly polarized and active antivaccine commentary [33]. There is growing evidence that social media can be an effective tool for public health promotion [15]. Digital health interventions can reach young adults regardless of geographical location and stigmatizing experiences with healthcare institutions. About 96% of 18–29-year-olds in the United States own a smartphone, making digital platforms highly accessible for delivering health interventions [34]. They have been shown to increase knowledge, self-efficacy, and motivation for change while also ameliorating distrust, fear, and stigma across a variety of health conditions. In the United States, digital storytelling workshops and personalized digital health interventions increased vaccine confidence by 25% and reduced vaccine hesitancy by 15%. Additionally, these interventions resulted in a 35% increase in trust toward healthcare providers and a 30% reduction in perceived stigma [34]. The dissemination of positive vaccination narratives and testimonials via social media has reinforced the appeal of vaccination. Analysis of social media sentiment showed that positive testimonials were associated with a 20% increase in positive vaccine-related posts, while negative sentiment towards vaccines decreased by 10% [30]. Collaboration with technology companies and social media platforms to flag and remove harmful content has been instrumental in reducing public exposure to misinformation. Fact-checking initiatives and promoting credible sources of information have enabled individuals to make informed decisions about vaccination [35]. Localized public health messages delivered via trusted community leaders and healthcare providers have also been demonstrated to be an effective method of communication [16]. It is of the utmost importance to ensure transparent communication, with open and honest messaging from trusted health authorities about vaccine safety, efficacy, and potential side effects. Public health messages emphasize guideline changes by providing context and explaining why the changes were made. Furthermore, new recommendations should be communicated repeatedly to reduce doubts [36,37]. 

Another crucial aspect is providing support to individuals in navigating the transition from uncertainty to vaccination. Contextually relevant information provided by a trusted healthcare provider has been an important factor in shifting many from hesitancy or resistance to cautious readiness. In a study involving focus groups, 44.4% of participants indicated that trust in their medical caretaker significantly influenced their decision to vaccinate. Additionally, personalized counseling and accurate information from healthcare providers resulted in a 72.2% increase in vaccine acceptance among the participants [38]. Primary healthcare providers are well placed to provide this information [39]. When talking to people hesitant to receive a vaccine, it is beneficial to prioritize values related to community care rather than facts about vaccine safety and efficacy. The use of plain language and active listening (ask–tell–ask) has been effective in this regard [25]. 

To facilitate optimal access, numerous strategies have been implemented, including establishing mass vaccination centers. These purpose-built facilities are designed to accommodate a high volume of vaccine recipients in a flexible timing and location [40]. Collaborations with community organizations have facilitated access to vaccination services in various settings, including homes, workplaces, community centers, care facilities for the elderly, and religious organizations. Community settings such as pharmacies, workplace clinics, and churches with extended hours and flexible scheduling have also been employed [41,42]. Establishing vaccination clinics near workplaces and shops has increased accessibility and reduced the barriers to vaccination, such as travel, scheduling, and the need for time off work. This intervention led to a 12.3% increase in vaccine uptake in nonmetropolitan counties and a 16.7% increase in large fringe metropolitan counties, highlighting the effectiveness of strategically placed vaccination sites [38,43]. 

Integrating vaccination services into national immunization programs, primary healthcare, and other relevant health services can reduce logistical barriers. Many countries are already integrating COVID-19 vaccination into their regular health services and exploring new entry points for vaccinating high-risk groups [44]. Mandating or imposing sanctions for non-vaccination has also been effective. In the United States, the intention to vaccinate was significantly higher when vaccination was required, with 86% of White respondents, 94% of Hispanic respondents, and 80% of Black respondents indicating that they would vaccinate when required, compared to 69%, 80%, and 56%, respectively, when vaccination was optional. Furthermore, an experimental study showed that vaccination intentions were significantly stronger in the required vaccination condition (M = 3.90, SE = 0.07) than in the freedom condition (M = 3.72, SE = 0.07) or the control for choice freedom condition (M = 3.77, SE = 0.07) [45]. Studies conducted in numerous countries have reported similar positive effects of incentives in the form of a COVID-19 pass on the willingness to be vaccinated [46,47,48,49]. Furthermore, the prospect of mandatory vaccination to obtain a Green Pass increased the likelihood of individuals accepting the vaccine, particularly when travel requirements were involved [50,51]. The introduction of COVID-19 passes has been associated with a notable increase in vaccination uptake in France (+13%), Italy (+10.7%), and Germany (+6.2%) [52]. Similar favorable results have been reported in different settings [53,54,55,56]. Financial incentives have facilitated the provision of this crucial vaccine program to at-risk groups sustainably and encouraged vaccine uptake in hard-to-reach groups [57]. Financial interventions, commonly referred to as “conditional cash lotteries”, provide financial gain contingent upon adopting a specific behavior. Most scientific evidence evaluating the impact of conditional cash lottery programs on COVID-19 vaccination rates originates from the United States, where several jurisdictions implemented cash lotteries to incentivize vaccination [58]. These programs have yielded mixed results in terms of increasing COVID-19 vaccination rates [59]. 

In conclusion, the most effective approach to enhancing vaccine acceptance is a multi-component one that combines effective communication, convenient access, targeted education, and community engagement. By applying these lessons and building upon successful strategies, public health efforts can effectively promote vaccine acceptance and safeguard communities from vaccine-preventable diseases.

## 5. Lessons Learned from Challenges and Barriers of COVID-19 Vaccination Campaigns

Despite the implementation of effective strategies implemented to reduce vaccine hesitancy, numerous other challenges hinder vaccination efforts. It is necessary to consider the logistical and economic barriers that have emerged. These barriers highlight areas where improvements can lead to more effective immunization programs. 

Logistical issues such as vaccine distribution, storage, and administration capacities are significant obstacles, especially in low-resource settings [60]. Economic constraints also affect vaccination programs, where funding shortages can limit the reach and frequency of campaigns. Socioeconomic and cultural barriers often intersect, as seen in rural versus urban disparities in vaccine uptake, influenced by access to healthcare facilities, information, and underlying healthcare infrastructures [61].

In light of these considerations, we analyze the main lessons and challenges for the future below. The first lesson underscored by the COVID-19 pandemic is the need for resilient supply chains. Ensuring a steady and reliable supply of vaccines requires coordination at global, national, and local levels. Investments in infrastructure, including cold chain logistics, are essential for the proper management of vaccination campaigns [62]. When the COVID-19 vaccine was developed, the first obstacle to the active immunization of people was the lack or absence of cold chain logistics, which was overcome by the advanced techniques of Pfizer and Moderna. Pfizer developed a single cryogenic package capable of transporting about 5000 vaccine doses at −70 °C in dry ice [63]. In this packaging, produced and shipped across Europe by Pfizer from Puurs in Belgium, the vaccine can be stored for up to 10 days. Additional dry ice can extend the storage period. Pfizer’s logistics system involves delivering these cryogenic boxes to individual regional hubs via couriers, where the receiving hubs transfer the vaccines into their super-freezers at −80 °C for secure storage [63]. On the other hand, Moderna avoided transporting its vaccine with dry ice, using refrigerated systems at −20 °C instead. These systems, consisting of phase change materials (PCM), thermoregulate the vaccine during transport. Moderna’s vaccine was distributed from the military base in Pratica di Mare to regional hospital hubs, where it is stored in freezers at −20 °C [63,64,65].

Moreover, partnerships with private sector logistics companies, the use of technology for tracking and inventory management, and decentralized distribution models enhanced reach and efficiency. Economically, COVID-19 highlighted the importance of innovative and flexible funding mechanisms. Collaborations between governments and private companies play a critical role in vaccine development, distribution, and administration, effectively mobilizing resources and expertise [66].

The challenges faced in past and future vaccine administration campaigns highlight the difficulties of reaching minority groups, such as rural populations and individuals with lower socioeconomic status. These groups often experience higher levels of vaccine hesitancy due to [67] various reasons, including historical mistrust in healthcare systems, cultural differences, and lack of access to accurate information. Therefore, it is essential to develop and implement targeted strategies to address these issues effectively [28].

One of the most impactful strategies is cultural competency training for healthcare providers. By understanding and respecting cultural differences, providers can improve communication and build trust with minority patients. This training helps ensure that all patients feel understood and respected, which can significantly enhance their willingness to receive vaccinations [28,68].

A systematic review of the literature reviewed five interventions to improve cultural competence in healthcare systems—programs to recruit and retain staff members who reflect the cultural diversity of the community served, use of interpreter services or bilingual providers for clients with limited English proficiency, cultural competency training for healthcare providers, use of linguistically and culturally appropriate health education materials, and culturally specific healthcare settings. The effectiveness of any of these interventions could not be examined [69]. 

Another crucial approach is community engagement. Partnering with trusted community leaders and organizations can play a pivotal role in spreading accurate vaccine information and organizing vaccination events in locations that are familiar and accessible to these communities. These partnerships can help break down barriers and make vaccination more accessible for hesitant groups [70].

Surveys have revealed a 10–20% reduction in vaccine hesitancy in populations receiving community-targeted information campaigns [71].

Tailored messages that address specific concerns of the community members can lead to a more significant reduction in hesitancy. Studies show that personalized engagement leads to a 25% higher likelihood of individuals deciding to vaccinate [72]. 

Phone outreach has proven to be a valuable tool, especially for communities that culturally prefer face-to-face or more personal interactions over the phone, such as Native Hawaiians and Pacific Islanders [73]. During the pandemic, the inability to communicate in person posed significant challenges. In Southern California, previous phone banking efforts for voter registration and Census outreach were repurposed by Native Hawaiian and Pacific Islander community-based and faith-based organizations to create “Wellness Phone Banking” [74]. This initiative aimed to check in with community members to determine their needs, such as prescriptions, food delivery, and other resources. This type of personal contact from trusted community members was beneficial for both gathering information and sharing resources [75].

In addition to addressing immediate needs, these efforts also highlighted the importance of addressing social determinants of health, which were exacerbated during the pandemic for many communities of color. In the more rural Central Valley, a free and confidential STOP COVID-19 San Joaquin Valley call line was established in both English and Spanish [76]. UC Merced students staffed this line to assist community members in making vaccination appointments. Similarly, in San Diego, community leaders relied on phone calls and text messages to set up vaccine appointments, manually entering information into the local immunization registry [74]. Despite the labor-intensive nature of this “high-touch” approach, it was necessary to overcome barriers faced by community members, such as navigating complex English-only websites or patient portals to make vaccine appointments [74].

For rural populations, specific challenges such as limited healthcare infrastructure and the long distances to vaccination sites need to be addressed. Implementing mobile vaccination units can greatly enhance accessibility and convenience for rural residents. Additionally, telehealth services can provide much-needed vaccine education and address concerns, effectively bridging the gap created by the physical distance from healthcare facilities [77].

Lower socioeconomic status groups face their own set of barriers, including limited access to healthcare, financial constraints, and a higher susceptibility to misinformation. To encourage vaccination among these individuals, financial incentives such as conditional cash transfers or lotteries can be particularly effective. Furthermore, launching accessible information campaigns that provide clear, simple, and multilingual vaccine information through various channels, such as social media and community centers, ensures that accurate information reaches all segments of the population [75].

By adopting these strategies, we can better support minority groups, reduce vaccine hesitancy, and improve overall public health outcomes. Addressing these challenges with thoughtful and inclusive approaches is key to the success of any vaccination campaign. By integrating these lessons, future vaccination campaigns can be more resilient, efficient, and equitable, ultimately leading to better public health outcomes and preparedness for future health challenges [78].

## 6. Overcoming Inequalities in Vaccination Access: Insights from the COVID-19 Campaign

Despite significant global efforts to ensure equitable vaccine distribution during the COVID-19 pandemic, substantial regional disparities persist, primarily influenced by variations in vaccine supply, healthcare infrastructure maturity, and political commitment levels [79]. These disparities highlight the necessity for region-specific strategies that account for the unique challenges and needs of each area to guarantee comprehensive vaccine coverage. Africa has faced notable vaccine shortages largely due to its dependence on donations and purchases through the COVAX initiative [80,81]. In stark contrast, Europe and North America have enjoyed more reliable access to COVID-19 vaccines, benefitting from well-established healthcare systems and pre-pandemic contracts that ensured an early and steady supply of large vaccine quantities [82]. These early agreements have been pivotal in preventing numerous COVID-19-related deaths. Research by Gozzi et al. indicates that timely vaccine access could have prevented over 50% of deaths in twenty lower-middle- and low-income countries [82].

Disparities in vaccine access are evident not only between countries but also within them, particularly between rural and urban areas. Rural regions frequently grapple with logistical issues, complicating the delivery and preservation of vaccines, moreover, rural areas may experience frequent power outages and lack advanced refrigeration technology [83]. Conversely, urban areas often benefit from advanced transportation infrastructure, and modern equipment which facilitates efficient vaccine distribution to local healthcare facilities [84]. These logistical challenges are further compounded by economic limitations and social determinants of health that exacerbate disparities. For instance, economic constraints can restrict the ability of such governments to enhance necessary infrastructure, and lower education levels may hinder public understanding of and trust in vaccines. Another important aspect that largely contributes to disparities in vaccine access is the vaccine procurement process, which influences not only vaccine availability but also the efficiency and fairness of distribution [85]. Procurement can be centralized, with supra-national entities negotiating purchases for an entire region, which can leverage economies of scale and lower costs. However, this might lead to distribution imbalances if regional needs are overlooked [85]. Decentralized procurement allows for customization of vaccine orders to better meet local health priorities but often at higher costs, which can disadvantage economically weaker areas [85].

Addressing disparities in vaccine access is a complex issue requiring multicomponent interventions [86]. Strategies that prioritize high-risk populations, expand vaccine distribution channels, and enhance outreach to underserved communities have been considered the most effective [86]. Individuals with disabilities, mental health conditions, and special needs have often required additional support to receive vaccinations, including disability-focused information, specific access and sensory requirements, and longer appointment times [86]. The availability of vaccines for carers at the same time has also been a significant consideration. In this perspective, governments must employ a range of approaches: robust epidemiological data, ensuring those most vulnerable are protected first; enhance the number and variety of vaccine distribution points, such as mobile units and community-based clinics; and lastly, involve multilingual education campaigns and partnerships with trusted community figures to increase vaccine acceptance and counteract prevalent misinformation [87,88]. Moreover, it is essential to ensure that resources are allocated efficiently, logistics are streamlined, and outreach strategies are effectively executed. Furthermore, harnessing technology and data analytics, like geographic information systems (GIS) and real-time monitoring, might help to identify areas with low vaccine uptake and adjust strategies accordingly [89].

By integrating these strategies, governments can address the deeply rooted disparities that impact vaccine access within their borders, ensuring that all segments of the population, especially the most vulnerable, have access to life-saving vaccines. This approach is vital for enhancing public health outcomes universally.

Through concerted effort involving international organizations, national governments, and private sector partners, implementing region-specific solutions that consider local contexts and needs is the only way to achieve truly equitable global vaccine coverage.

## 7. Implications for Future Public Health Strategies

**Figure 1:**
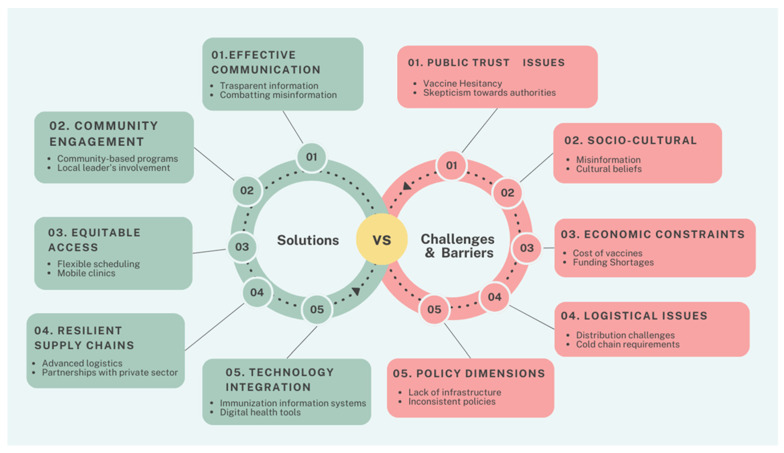
Challenges, barriers, and possible solutions learned from the COVID-19 pandemic.

The COVID-19 pandemic has underscored the importance of adaptable and resilient public health systems capable of rapid response to emerging health crises. The lessons learned can inform future vaccination campaigns and broader public health initiatives [2]. Figure 1 summarizes the challenges, barriers, and solutions discussed in this paper. Strengthening global cooperation, enhancing public trust, and ensuring equitable access to healthcare resources are fundamental to improving public health outcomes. In particular, investments in public health infrastructure, including advanced cold chain systems and robust distribution networks, are vital for managing future health emergencies [90]. For instance, Pfizer’s development of cryogenic packaging capable of transporting large quantities of vaccines at ultra-low temperatures exemplifies a successful logistical intervention that can be replicated in future campaigns [91]. Moreover, enhancing health literacy and integrating vaccination services into regular healthcare provisions can further bolster public health resilience [92]. Telehealth services and mobile vaccination units have demonstrated significant improvements in vaccine accessibility. For example, the introduction of a mobile vaccination unit into a neighborhood, in the United Kingdom, increased the number of first vaccinations conducted by 25% [77]. At the same time, digital tools such as social media also have an important role. A systematic review of 25 articles identified several facilitators associated with vaccine promotion through social media use. Facilitators included increased efforts by social media companies to reduce misinformation and the use of social media to disseminate public health information and promote vaccination [93]. Furthermore, the pandemic has underscored the importance of international collaboration in addressing global health challenges [94]. Sharing resources, knowledge, and best practices can help build a more cohesive and effective global health response system. The success of mRNA vaccines during the COVID-19 pandemic highlights the potential of innovative vaccine technologies [7]. In this perspective, continued research and development in this area can lead to more effective and rapidly deployable vaccines for various infectious diseases.

The actionable insights derived from this analysis should inform policy and practice, ensuring that public health strategies are evidence based and context specific. By applying these lessons, public health efforts can more effectively promote vaccine acceptance and protect communities from preventable diseases. 

## 8. Conclusions

The analysis of the COVID-19 pandemic’s impact on vaccination strategies highlights critical lessons and outlines effective measures for enhancing vaccine acceptance and uptake. The rapid development and deployment of COVID-19 vaccines showcased the potential of scientific innovation, particularly with the advent of mRNA technology. However, the unprecedented speed also fostered vaccine hesitancy driven by misinformation, distrust, and logistical challenges. Addressing these issues requires a multifaceted approach that combines effective communication, community engagement, and logistical solutions. 

In conclusion, the COVID-19 pandemic has provided invaluable insights into the complexities of vaccination promotion and public health strategy. By building on these lessons and implementing comprehensive, multi-component approaches, public health systems can enhance vaccine uptake and better safeguard global health.
